# Feeding strategies alter gene expression of the calpain system and meat quality in the *longissimus* muscle of Braford steers

**DOI:** 10.5713/ajas.19.0163

**Published:** 2019-08-03

**Authors:** María Sumampa Coria, Pablo Sebastián Reineri, Dario Pighin, María Guadalupe Barrionuevo, Pedro Gabriel Carranza, Gabriela Grigioni, Gustavo Adolfo Palma

**Affiliations:** 1 Animal Production and Reproduction Laboratory, NOA Institute of Bionanotechnology (INBIONATEC), Villa El Zanjón, Santiago del Estero, G4206XCP, Argentina; 2 National Council of Scientific and Technical Research (CONICET), Buenos Aires, C1033AAJ, Argentina; 3 Faculty of Agronomy and Agribusiness (FAyA), National University of Santiago del Estero (UNSE), Santiago del Estero, G4206XCP, Argentina; 4 Santiago del Estero Agricultural Experimental Station, Francisco Cantos Experimental Field, INTA, La Abrita, Santiago del Estero, G4206XBK, Argentina; 5 Food Technology Institute, Agroindustry Research Centre, INTA, Castelar, Buenos Aires, B1712, Argentina; 6 Faculty of Agriculture and Food Sciences, Moron University, Morón, Buenos Aires, B1708JPD, Argentina; 7 Santiago del Estero Centre of Research and Transfer (CITSE), Villa El Zanjón, Santiago del Estero, G4206XCP, Argentina

**Keywords:** Beef Cattle, Cattle Feeding, Calpain System, Meat Quality, Meat Tenderness

## Abstract

**Objective:**

The aim of the present study was to determine the effect of supplementing pasture-finished steers with corn silage on the expression level of the calpain system proteins and beef tenderization.

**Methods:**

Thirty Braford steers grazing on summer pasture were used for the study. For 120 days fifteen animals were supplemented with corn silage at 1% of body weight per head per day (Suppl) whereas the remaining 15 steers only received pasture (Contr). Carcass and meat traits were evaluated and compared between groups. Gene expression and activities of proteases (calpain 1 and calpain 2) and inhibitor (calpastatin) were measured using real-time polymerase chain reaction and casein zymography.

**Results:**

Carcass and meat traits were significantly different between feeding systems. Supplemented steers showed higher hot carcass weight (p<0.01), fat content (p = 0.02), and Warner-Bratzler shear force (p = 0.03). Furthermore, the control group showed higher protease:inhibitor ratios, at mRNA (p = 0.01) and protein levels (p<0.10). Warner-Bratzler shear force and mRNA calpains:calpastatin ratio were associated in both feeding systems (p<0.01).

**Conclusion:**

Based on the results obtained in the study, beef tenderness differences among finishing strategies could be modulated through differential expression of the calpain system proteins.

## INTRODUCTION

An important challenge of livestock production systems is to satisfy the demand for high quality meat. Tenderness is one of the most important factors affecting consumer’s satisfaction for beef palatability. This quality trait depends on many factors such as breed, age, amount and solubility of collagen, intramuscular fat (IMF) content, *post mortem* pH decline curve, and fibre type characteristics [1]. Genetic markers in calpain system have been associated with beef tenderness [2]. In skeletal muscle two proteases, calpain 1 and calpain 2, as well as their inhibitor, calpastatin, have been described in detail [3]. The mRNA and protein levels of this calpain system have often been examined in an attempt to understand their role in myofibrillar protein metabolism and meat tenderization. Generally, decreased calpain expression or increased calpastatin expression are associated with tougher meat. It has been reported previously that feeding strategies could modify protein expression of the calpain system. Therkildsen showed that activity of calpain-1 decreased after long-term feeding restriction [4]. It has also been reported that differences in the composition of nutritional diet modified calpastatin content in bovine skeletal muscle [5]. However, to our knowledge, the influence of feeding strategies on calpain gene expression has never been determined in cattle.

In order to produce high-quality meat is necessary to as sess animal production-related variables such as genetics and management that regulate muscle gene expression [6,7]. It has been established that feeding strategies can modulate muscle protein turnover, muscle energy levels during slaughter practices and water holding capacity (WHC), which can result in modifications in meat tenderness, the pH/T decline curve and sensory characteristics of the meat [8]. Therefore, the aim of the present study was to assess the effect of supplementing pasture-finished steers with corn silage on the expression level of the calpain system proteins and beef tenderization.

## MATERIALS AND METHODS

### Animals and sampling

The present study was conducted with animals from a commercial breeding herd in Santiago del Estero, in the northwest of Argentina (coordinates 27°17′34.3″S - 62°15′14.1″W). Animal handling and experimental procedures were in accordance with Handbook of Procedures for Animal Welfare of the National Service of Animal Health of Argentina (Servicio Nacional de Sanidad Animal, SENASA). The steers used in the present study had an average age of 22 months at the beginning of the experiment and they had been raised under extensive grazing on tropical pastures. The stocking rate was 1.5 animals/ha and the animals were moved between paddocks every 4 months. Thirty Braford steers grazing on summer pasture (*Megathyrsus maximus*) were chosen and divided randomly into 2 experimental groups: 15 animals were fed *ad libitum* pasture and supplemented with corn silage (1% of body weight per head per day [DM basis] during 120 days) (Suppl) whereas the other 15 steers were fed *ad libitum* similar pasture in different paddocks without corn supplement (Contr). Animals were weighed without fasting every 14 days. Corn silage was provided once a day early in the morning and the amount was adjusted according to the predicted average weight of the animals in the paddocks. Animals of both diets had free access to water. After allocation to feeding strategies, animals were placed randomly in paddocks in groups of 5 animals (3 paddocks per feeding strategy).

Herbage samples for assessment of the nutritive value were collected via handclipping. Samples were taken on a diagonal transect within each paddock, with clip samples taken every 5 steps. Corn silage samples were collected at the beginning, middle and end of the unloading process for each paddock. All samples were dried in a 60°C forced-air drying oven and used for measuring crude protein, acid detergent fibre and neutral detergent fibre content. The chemical composition of feeds offered is given in Table 1.

Animals were slaughtered when they were in average 26 months of age. All steers had an average back fat thickness of at least 3 mm, as determined *in vivo* by ultrasound. Steers were transported to a licensed commercial abattoir by the same driver, following the same route, and they were kept in covered yards with feed deprivation, but with free access to water. After the fasting period, steers were stunned using a captive bolt pistol and slaughtered according to standard commercial procedures. Animals were slaughtered in the same day after mixing groups to avoid peri-slaughter effects. Carcasses were suspended through the Achilles tendon and they were not electrically stimulated.

Carcasses were weighed immediately after slaughtering (hot carcass weight, HCW). Classification of conformation and fatness degree were based on visual assessment by four trained and experienced evaluators. The Argentine Grading System (National Meats Board, Resolution J-378/73 of SAGPSyA) is structured per animal categories classes and subclasses of quality evaluated. Steers carcass conformation assigned by the meat grader according to the Argentine Grading system are based on visual assessment of muscle mass development, where a higher number indicates better conformation, where JJ is excellent (7), J is very good (6), U is good (5), U2 is medium (4), N is fair (3), T is regular (2), and A is poor (1). Fat degree was a subjective measurement based on the observation made by experts to determine the thickness, accumulation and distribution of adipose tissue that covers the carcass. The different degrees of fatness are given by the relation of the muscular masses with the covering fat: 0 (slight), 1 (modest), 2 (moderate), 3 (abundant), and 4 (excessive).

Samples of approximately 500 mg of the *longissimus thoracis et lumborum* (LTL) muscle were obtained within 20 min of exsanguination, frozen in liquid N_2_ and stored at −80°C until molecular analysis. In abattoir carcass pH and temperature were measured 45 min and 48 h *post mortem* in the LTL muscle, at a point located within the interval between the 12th and 13th rib, using a Testo 205 pH meter (Testo AG, Lenzkirch, Germany). After slaughtering and following a 48 h cooling period between 1°C and 5°C, the block of steaks between the 9th and 13th rib was removed from each left half carcass. The block was deboned, vacuum-packed and frozen at −18°C. Prior to thawing, each block was subsampled with an electric saw in steaks of 2.54 cm width, vacuum packaged and kept at −18°C. Analytical meat determinations were performed at the Instituto Tecnología de Alimentos, Instituto Nacional de Tecnología Agropecuaria (INTA), Castelar, Buenos Aires.

### Rib eye area and marbling

The cross-sectional area of the eye muscle (rib eye area, REA) was determined in the LTL muscle area at the 12th rib. *Longissimus* REAs were drawn on tracing paper and measured using a Koizumi digital planimeter KP-92N (PLACOM, Tokyo, Japan) and a millimetric ruler. Once samples were thawed, the degree of marbling was determined by visual appraisal, and compared with the United States Department of Agriculture standard set of coloured pictures. The marbling score was converted into a numerical code as follows: 0.0, devoid; 0.5, practically devoid; 1.0, traces; 1.5, slight; 2.0, small; 2.5, modest; 3.0, moderate; 3.5, slightly abundant; 4.0, moderately abundant; 4.5, abundant; 5.0, excess.

### Water holding capacity and cooking loss

The WHC was determined in duplicate following the filter paper press methodology. Briefly, this technique involves compression of 0.3±0.02 g of sample, using a filter paper (Munktell Filter, Quality 1003, Size 90 mm) and measuring the wetted area. The WHC was expressed as the percentage of meat free juice expelled (WHC = meat area/total liquid infiltrated area ×100). This procedure assumes that the area of the ring of expressed juice absorbed by the filter paper is related to the amount of meat free water.

The cooking loss (CL) was determined through measur ing the sample weight before and after heat treatment, after cooling for 20 min at room temperature. Steaks were subjected to heat treatment with dry heat cooking (oven temperature, 170°C; temperature of the thermal center of the samples, 71°C). The CL was reported as the percentage of weight loss with respect to the initial weight of the sample.

### Muscle colour and pH

Meat colour was assessed using a Commission Internationale d’Eclairage Lab System, which provides values for colour components: L* (black–white, lightness) and the chromatic coordinates a* (+ to −, from red to green) and b* (+ to −, from yellow to blue). Measurements were carried out using a Minolta CR-400 chromometer (Konica Minolta Sensing, Inc., Bergen, NJ, USA). The instrumental conditions were: artificial D65 illuminant and 2-degree standard angle observer. Determinations were carried out with 2.5 cm thick steaks. Each sample was allowed to bloom for 45 min prior to the first measurement, and six scans of each steak were averaged for statistical analysis.

Muscle pH was measured using a Testo 205 pH meter (Testo AG, Germany). Each measurement was performed in triplicate, taking the mean values as the assay result.

### Instrumental tenderness analysis

Instrumental tenderness was measured using the Warner-Bratzler test, assessing the resistance to shear force cut in cooked meat. Once thawed, meat cuts were deboned, weighed and placed in a pre-heated electric grill until they reached a final internal temperature of 71°C. Cooked steaks (2.5 cm thickness) were weighed and cooled down to <10°C overnight. Samples were obtained by cutting 1.3 cm diameter cores from each steak parallel to the muscle fibre orientation and sheared once across the middle (in direction perpendicular to the fibres) using a 1.016 mm WB probe in a TA.XT Plus Texture Analyzer (Stable Micro Systems Ltd, Surrey, UK). The highest peak in Newton (maximum shear force) was measured in 7 to 10 cores per sample.

### Intramuscular fat and collagen content

To determine IMF content, two aliquot samples of 5 g each, trimmed of external fat, were minced, dried and extracted in a Tecator equipment using hexane as extraction solvent according to official methods.

Total collagen content was determined by the methodology described by Kolar [9]. Briefly, 4 g of muscle were hydrolyzed with sulfuric acid. A solution of hydroxyproline, made from a stock solution (600 μg/mL), was used to obtain a standard curve. Samples and standard solution were added to an oxidant solution (chloramine-T) and the formation of a reddish purple complex with 4-dimethylaminobenzaldehyde was read at 560 nm using a NanoDrop 2000c UV-Vis Spectrophotometer (Thermo Scientific, Barrington, IL, USA).

### Calpain and calpastatin activity

#### Protein extraction and quantification

Protein was measured in all samples. From each frozen sample (−80°C), duplicates of 200 mg of meat were homogenized in 1 mL of extraction buffer (100 mM Tris-HCl; 10 mM ethylenediaminetetraacetic acid (EDTA); 0.05% β-mercaptoethanol; pH 8.3) at 13,500 rpm using a D-160 Handheld Homogenizer (Dragon Lab, Beijing, China) and centrifuged for 30 min at 4°C at 8,800×*g*. An aliquot of the supernatant was mixed with glycerol at a final concentration of 30% and stored at −80°C until calpain activity was analyzed. Concentration of proteins was determined with the Quick Start Bradford protein assay (Bio-Rad, Hercules, CA, USA), following the standard micro plate protocol. For calpastatin activity, another aliquot of supernatant was heat treated in a water bath at 100°C during 5 min, centrifuged during 5 min at 20,000×*g*, and stored at −80°C until analysis.

#### Casein zymography method

Calpain and calpastatin activity were determined using the casein zymography method. Briefly, casein (0.2%, wt/vol) was incorporated in 12% separating gels (75:1 ratio of acrylamide and bisacrylamide, 375 mM Tris-HCl, pH 8.8), and 5% gels (75:1 ratio of acrylamide and bisacrylamide, 125 mM Tris-HCl, pH 6.8), free casein stacking gel were used. Tetramethylethylenediamine (0.05%, vol/vol) and ammonium persulfate (0.05%, vol/vol) were employed to catalyze the polymerization reaction. Sample buffer (225 mM Tris-HCl, pH 6.8, 30% glycerol, 1.12% β-mercaptoethanol, 0.03% wt/vol bromophenol blue) was added to the supernatant equivalent to 40 μg of the total proteins reaching a final volume of 20 μL. The casein gels (1 mm) were prerun at 0.02 A per gel for 15 min at 4°C, with a running buffer containing 25 mM Tris-HCl, 0.05% β-mercaptoethanol, 192 mM glycine, and 1 mM EDTA (pH 8,3) before samples were loaded into the wells. The gels were then run at 0.02 A per gel for 3 h at 4°C, removed, and incubated at room temperature for 1 h in 50 mM Tris-HCl, 0.05% β-mercaptoethanol, and 4 mM CaCl_2_ (pH 7.5) under gentle shaking (three changes of buffer). This step was followed by incubation for 16 h in the same buffer at room temperature, prior to staining with Coomassie blue (R-250) for 1 h and destaining with 20% methanol and 7% acetic acid for 2 h. Clear bands indicating calpain activity were quantified comparing the density of each band with the reference standard of each gel. Before statistical analysis, calpain band densities were calculated as a percentage of a standard included in each gel. The standard was a sample of pasture-finished steers taken randomly in the same experiment.

### Free calcium concentration

Determination of free calcium was carried out according to Pomponio and Ertbjerg [10] with some modifications. Muscles (12 g) held at −80°C were removed from frozen storage and kept at room temperature for 10 min before they were finely diced, and then kept on ice again for 20 min before they were centrifuged at 20,000×*g* during 20 min. An aliquot of the supernatant was mixed with 4 M KCl. Finally, calcium concentration was determined in triplicate using the Ca-Color AA kit (Wiener Lab, Rosario, Argentina) following the manufacturer’s instructions. Absorbance was read at 570 nm in a NanoDrop 2000c UV-Vis spectrophotometer (Thermo Scientific, USA).

### RNA extraction, reverse transcription and quantitative polymerase chain reaction

Total RNA was extracted from 50 mg of muscles using TriReagent (Sigma, St. Louis, MO, USA) according to the manu-facturer’s protocol. Total RNA, eluted with sterile distilled water, was stored at −80°C. Concentration and purity of extracted RNA were determined using a NanoDrop 2000c UV-Vis spectrophotometer (Thermo Scientific, USA). RNA integrity and absence of genomic DNA were analyzed by agarose gel electrophoresis, and compared with base pair standards.

To ensure genomic DNA was removed from RNA, ampli fication grade deoxyribonuclease I (AMPD1, Sigma, USA) was incubated with RNA following the manufacturer’s instructions. Reverse transcription was performed using SuperScript III Reverse Transcriptase (Invitrogen, Carlsbad, CA, USA). Every 20 μL of reaction contained 1 μL of Oligo dT (50 μM), 1 μL of dNTPs, 4 μL of 5X First Strand Buffer, 1 μL of 0.1 M DTT, 40 units of RNAsin (Genbiotech, Buenos Aires, Argentina), 200 units of SuperScriptIII reverse transcriptase, a volume of RNA (50 ng/μL RNA), and nuclease-free water to complete the final volume. The mixture was held 1 h at 50°C and 15 min at 70°C prior to cooling on ice.

Messenger RNA levels of calpains (1 and 2) and calpastatin genes were determined by reverse transcription and quantitative polymerase chain reaction (RT-qPCR) using specific primers designed with the Primer-BLAST tool (Table 2). Primers were previously validated for adequate primer efficiency, and specificity of their PCR products was confirmed by 1.5% agarose gel electrophoresis. The PCR, with a final volume of 10 μL, containing 3 μL of cDNA template (diluted 1:3), 0.25 mM of forward and reverse primers and 5 μL of iTaq Universal SYBR Green Supermix (Bio-Rad, USA) were performed in a CFX96 real-time PCR detection System (Bio-Rad, USA). The PCR program consisted of an initial step of 5 min at 95°C, followed by 50 cycles of 30 s at 95°C, 30 s at 58°C for annealing and 30 s at 60°C for extension. At the end of each PCR run, melt curve analysis was performed for all genes to ensure single product amplification and exclude any possible interference of dimers. No-template and no–reverse transcription controls were included as negative controls and to exclude genomic DNA contamination.

### Statistical analysis

Student’s t-test was used for independent samples to compare supplemented and control groups using animal as experimental unit. Means of carcass traits, measured during the slaughter period in each left half carcass and meat quality traits, measured in the *longissimus dorsi* muscle, were compared using Infostat software [11]. For all assays, the level of significance was set at 0.05.

Gene expression analysis was measured as proposed by Mberema et al [12]. Fold differences in gene expression between Suppl and Contr groups were calculated according to the efficiency corrected method. For each sample, arithmetic mean of its technical replicates’ Ct values were converted into relative quantification values (Q) following the delta-Ct formula Q = E^−ΔCt^, where E is the efficiency of a the target gene and ΔCt is the difference between the Ct mean value of target gene in control and sample. The efficiency was calculated and verified using the standard curve obtained by amplification of serial dilution of the pooled cDNA. The Q value obtained for each sample was normalized by dividing with the geometric mean of the expression of two reference genes in the given sample. The reference genes used in the present study were the ribosomal protein large protein 0 and glyceraldehyde-3-phosphate dehydrogenase listed in Table 2. The entire study was carried out following the guidelines for minimum information for publication of quantitative real-time PCR experiments.

Proteases and inhibitor ratios were calculated at mRNA and protein levels. Data from ratios were transformed into a new variable using range transformation, this function assigns to the original data the position that each one occupies in the series ordered in ascending order. Spearman rank correlation coefficients between ratios were calculated and residual correlations among meat traits and proteases: inhibitor ratios were estimated for groups treated combined or individually.

## RESULTS AND DISCUSSION

### Characteristics of slaughtered animals

The effect of the different feeding systems on the characteristics of slaughtered animals is shown in Table 3. The average daily gain (ADG, kg/animal) of Braford steers differed between corn silage and pasture finished animals (p<0.0001), resulting in an average weight of the supplemented and pasture-fed steers at the end of the finishing period of 472.62 and 455.12 kg, respectively (p = 0.0499). As was previously described, when feed is not restricted, Braford animals usually have significant ADG because they have a great potential for muscle accumulation and their consumption is higher per unit weight. Thus, supplemented-fed animals have heavier and fatter carcasses than forage-fed animals when grown for a constant time period. These results are consistent with findings by other authors who compared pasture-fed animals and grain-supplemented animals (1% body weight) [13,14]. In general, steers supplemented received higher nutrient supply, which determined the difference observed in final body weight in relation to those that were not supplemented. In the present study, the supplementation offered to the supplemented group during 120 days allowed animals to achieve higher daily gains and improve HCW.

Moreover, conformation scores tended to be U2 or U (6 or 5) indicating a better conformation of carcasses finished on pasture than corn-supplemented ones. Carcasses from supplement-finished animals had greater fat depth and fat degree (p = 0.0496 and p = 0.0001, respectively) than pasture-finished animals. These results were in agreement with previous studies, where finishing-feeding effect was evaluated, suggesting that finishing strategies had an effect on carcass conformation and fat grading [15]. Finishing fat is important because it determines when cattle can be marketed. In general, higher carcass weight produces higher muscle thickness and fat deposits. Supplemented steers presented approximately 4.45 mm of fat; and pasture finished carcasses presented approximately 3.46 mm of fat, which is the lower limit for marketing carcasses in more demanding markets, for correct carcass chilling, and for adequate meat color [15].

Feeding did not alter (p = 0.1746) pH values measured in abattoir at 45 min after slaughter, but mean pH at 48 h was lower in carcass from supplemented group (p = 0.0004). It is well known that supplementing grazing cattle increases muscle glycogen storage, which in turn reduces muscle pH after 24 h [8]. Despite changes in pH after 48 h previously mentioned, no dark, firm, dry characteristics were found. Therefore, the beef obtained was considered appropriate for this study.

### Meat quality

Meat quality characteristics such as CL, WHC, pH, colour, REA, marbling score, Warner-Bratzler shear force (WBSF), IMF, calcium and collagen content are given in Table 4.

The values of CL and WHC were significantly different between feeding systems, resulting in meat with higher WHC (p<0.0001) and lower CL (p<0.0006) in the supplemented group compared with pasture alone, respectively. These values are higher than those obtained in other study performed in Braford steers [14]. Nevertheless, in the present study meat was frozen and it is known that the cell membranes are damaged during this process, which results in a lower WHC and a higher CL [16].

Objective colour determination of the *longissimus* muscle is also shown in Table 4. Although some authors found differences associated with treatments involving different carcass weight and variations in the chilling rate [17], there were no differences (p>0.05) in longissimus L*, a*, or b* values between treatments in the present study. Other researchers did not find significant effects of different forage/concentrate ratio diets during finishing on muscle colour [13–18]. Diet characteristics do not have capital relevance on meat colour, probably due to transformation processes that take place in the rumen.

The WBSF values were higher for beef from the corn-sup plemented group than for beef from the pasture-only group (p = 0.0306). Several studies comparing forage versus concentrate finishing of beef have provided mixed results regarding meat tenderness. Some studies found a negative effect of forage finishing on meat tenderness [19], while others showed that pasture-fed beef can be produced without deleterious effect on meat quality, including tenderness [20]. The high variability of data reported could be attributed to factors like different breeds, diets, pre-slaughter treatments, and methodologies employed.

The management regime associated with the different pro duction systems is an important factor that usually plays a significant role in determining collagen characteristics and beef texture. In agreement with other study that have assessed the influence of diet on meat quality and collagen properties, in the present study the collagen content did not differ between the feeding groups [21].

Calcium greatly affects both activity and autolysis of cal pains [3], which motivated us to determine the free calcium content in beef samples in the present study. As can be seen in Table 4, the calcium content did not change significantly with the feeding regime. In contrast, other authors found differences in calcium associated with vitamin supplement [22, 23]. In those studies, the vitamin supplement provided intended to increase serum calcium, thus creating an influx of calcium into the skeletal muscle. This would cause a decrease in calpain-1 mRNA levels or activity, or a potential increase in calpastatin activity. These effects were not observed in the present study.

As expected, marbling scores were higher in supplement ed animals. Similar results have been reported by Fumagalli et al [24] in an assay carried out with Braford steers supplemented with corn grain (0.7% and 1.2% body weight). Beef from steers reared only with pasture had lower IMF content than meat from supplemented steers (p = 0.0174). These results are in concordance with other authors, who found that pasture-finished beef was leaner than grain-finished beef [20]. Hence, the higher levels of IMF found on corn-finished beef are consistent with the values of marbling in the present study, which displays a positive association between the energy level of the diet and the degree of IMF deposition.

### Calpain system

Gene expression data were presented in Table 5 as fold change ratio having as reference the pasture-finished group. The down-regulation of proteases, calpain-1 (*capn1*) and calpain-2 (*capn2*), in supplemented animals would lead to a reduction in proteolysis of myofibrillar proteins resulting in a poor tenderization, supported by higher shear force values in supplemented meat in our study. Conversely, higher calpains levels in grass-finished animals imply high proteolytic activity in the muscles of these animals and consequently tender meat.

Giusti et al [25] suggested that the slightly lower tender ness of meat is probably not the result of a lower expression of genes encoding proteases (*capn1* and *capn2*), but it is more likely related to an increased calpastatin (*cast*) expression. In agreement, the up-regulation of the inhibitor could contribute to explaining the different WBSF values previously described (Table 4). Previously it has been reported that feeding strategies could modify protein expression of the calpain system [4,5]. Nevertheless, increases in mRNA and/or protein expression are not necessarily correlated with increased activity since the net activity of calpains depends on the calpain:calpastatin ratio. Moreover, it has been proposed that this ratio could determine the degree of *post mortem* proteolysis and intramuscular differences in protein turnover, assuming that it could account for *post mortem* proteolysis and meat tenderization [26]. In the present study, both ratios (*capn1*:*cast* and *capn2*:*cast*) presented differences between treatments. Protease: inhibitor ratios were higher in Contr. group, suggesting that feeding systems may influence expression of both proteases and their inhibitor and, consequently, affect beef tenderness (Table 5).

Data of activity of pre-rigor calpain system proteins in pas ture- and corn silage-finished animals are presented in Table 5. Muscle from supplemented animals had lower levels of calpain 1 activity (CAPN1) (p = 0.0397) compared with pasture finished animals. On the other hand, calpain 2 activity (CAPN2) did not differ significantly between treatments (p = 0.1186). Total calpastatin activity (CAST) increased in corn-finished animals (p = 0.0099). When calpain:calpastatin ratios were analyzed, both CAPN1:CAST and CAPN2:CAST tended to be higher in muscle from pasture-finished cattle (p = 0.0812 and 0.0742, respectively). This would indicate a tendency of higher calpain activity for every unit of calpastatin activity in the LTL muscle from pasture-finished animals. A higher calpain:calpastatin ratio would predict an increased protein turnover in the living muscle and would promote an increased *post mortem* proteolysis in beef from these animals. Consequently, it can be assumed that grass-fed beef would have a faster and/or a greater development of tenderness when compared with supplemented beef. In contrast to our results, other authors found that activity of calpains and their inhibitor was not modified by restrictive and realimentation strategies in bull calves [26]. Thomson et al [27] found in an experiment with steers exposed to different durations of restricted and *ad libitum* feeding, that a response of the calpain system depended on the breed. In summary, the response of the calpain system depends on the genotype and age of the animals, the muscle studied, the degree of restricted feeding compared with *ad libitum* feeding and the composition of the supplement.

### Calpain system and meat traits

The ratio between protease and inhibitor was calculated at mRNA and protein level and results of correlation analysis between ratios are given in Table 6. Several authors have found similar associations between mRNA expression of calpain and calpastatin genes and the respective activities [4,28]. Furthermore, a correlation analysis between ratios and meat traits was carried out. The protease:inhibitor ratios, at mRNA and protein levels, were significantly correlated with carcass and meat quality traits such as HCW (p<0.01), fat degree (p<0.01), CL (p<0.01), and WHC (p<0.01) (Table 7). Calpain:calpastatin ratios, at protein level, were significantly correlated with IMF (p<0.01) (Table 7). Similar to our results, a higher carcass weight in bull calves was associated with lower calpain levels [4], or with higher inhibitor expression in cows and fetuses [5]. In addition, HCW in Angus bulls was correlated with calpain 2 activity (−0.519, p<0.01) [29]. It is generally accepted that WHC of bovine muscle is negatively correlated with IMF and that variations in the WHC may be due to differences in *post mortem* degradation of proteins [30]. Nevertheless, further biological or functional evidence is necessary to confirm the genetic effects of the calpain system on fat content.

The mRNA protease:inhibitor ratios were also correlated with WBSF (p<0.01) (Table 7). Pringle et al [31] demonstrated that the ratio between calpain activity and protease inhibitor were correlated with WBSF in Angus and Brahman crossbred steers feeding corn-protein supplement diet (−0.260, p<0.05 and 0.440, p<0.01, respectively); even though the ratio was expressed inversely, the association was similar. A stronger correlation was found between the shear force and the 135 kDa calpastatin isoform 24 h *post mortem* in Friesian calves (0.620, p<0.01) [32] or calpastatin activity in *Bos taurus* crossbred steers (0.555, p<0.01) [33]. The calpain:calpastatin ratio has been found to be a good indicator of the proteolytic capacity of the muscle and hence it should be related to the degree of *post mortem* tenderization that occurs in the muscle. The decreased level of calpastatin results in an increase in calpain-mediated proteolysis and consequently, meat tenderness improves. The calcium concentration has also been reported to play an important role in the activity of this proteolytic system, and although other authors found associations between calcium content and the calpain system proteins, an association between these traits could not be found in the present study [23].

To our knowledge, this is the first report that reveals a cor relation between feeding strategies, tenderness and gene expression in Braford cattle. Our results indicate that determination of the gene expression of the calpain system proteins could be used as a genetic test for screening individuals for tender meat. The meat industry would benefit from the ability to genetically select animals that are superior and consistent for meat quality traits.

Supplementing pasture-finished steers with corn silage re sulted in faster growth rates and higher carcass fat degree. Beef quality of corn-finished animals displayed higher fat content, WHC and WBSF, and smaller CL. Although extensive production systems-mainly based on pastures- are traditionally associated with inferior meat quality attributes, the present study did not show clear changes in meat quality attributes between finishing strategies. Moreover, pasture finishing increased beef tenderness under the assay conditions. Our results indicate that differences in tenderness among finishing strategies could partially be explained by variations in the expression of the calpain system at mRNA level; corn silage supplement decreased the amount of calpains and increased expression of calpastatin in *longissimus dorsi* muscle. A better understanding of the molecular basis of meat tenderization is particularly useful to the meat industry and it may allow modifications of pre-slaughter handling practices and *post mortem* treatments to improve meat quality. Furthermore, results of the present study suggest that the proteases and the inhibitor studied seem to have the potential for an easy, fast and economical way of evaluating beef quality.

## Figures and Tables

**Table 1:** Mean values for chemical composition of finishing diets fed to Braford steers (120 days)

Chemical composition	Finishing treatments	

	Pasture	Corn silage
CP (%)	9.13±0.65	20.15±0.85
ADF (%)	14.16±0.15	30.40±0.56
NDF (%)	24.62±0.32	50.85±0.38
OMD (%)	60.00±0.89	81.25±0.91

All values on dry matter basis. The values are means±standard deviation.

CP, crude protein; ADF, acid detergent fibre; NDF, neutral detergent fibre; OMD, organic matter digestibility.

**Table 2:** Oligonucleotide sequences used for quantitative reverse transcriptase polymerase chain reaction assays

Assay1)	Primers2)
*capn1*	F: GAGGGCCGAGGAGATACCGTGAAT
	R: CCCGCTGCTTCTGCACTTGTG
	A: 112; AN: NM_174259; E: 0.95
*capn2*	F: TGACCCAAACTGGGCATCTGTCTA
	R: AAACAAGCTTGGGTGGTTTCCCTG
	A: 161; AN: NM_001103086; E: 0.99
*cast*	F: GCCACACCCAGGAGCATGTCAGT
	R: AGACTTGCTGGCTGACGCAGAG
	A: 182; AN: NM_001030318; E: 1.04
*rplp0*	F: CAACCCTGAAGTGCTTGACAT
	R: AGGCAGATGGATCAGCCA
	A: 226; AN: NM_001012682; E: 1.10
*gapdh*	F: AGATGGTGAAGGTCGGAGTG
	R: GAAGGTCAATGAAGGGGTCA
	A: 117; AN: NM_001034034 E: 0.97

1) *capn1*, calpain 1; *capn2*, calpain 2; *cast*, calpastatin; *rplp0*, ribosomal protein large protein 0; *gapdh*, glyceraldehyde-3-phosphate dehydrogenase.

2) Details of specific primer sets used: F, forward primer sequence (5′ to 3′); R, reverse primer sequence (5′ to 3′); A, amplicon length in base pairs; AN, accession number; E, efficiency in quantitative polymerase chain reaction.

**Table 3:** Growth performances and carcass characteristics of cattle finished for 120 days on pasture supplemented with corn silage (Suppl) or pasture alone (Contr)

Variable	Finishing treatments1)		p-value

	Suppl	Contr	
ADG (kg/d)	1.01±0.10	0.85±0.06	<0.0001
LW (kg)	472.62±18.38	455.12±16.44	0.0499
Fat depth (mm)	4.45±0.62	3.46±0.75	0.0496
HCW (kg)	298.53±17.08	264.40±15.34	<0.0001
Conformation score2)	5.80 ±0.41	6.00±0.00	0.0824
Fat degree2)	1.67±0.48	1.00±0.00	0.0001
pH 45 min3)	6.67±0.27	6.81±0.24	0.1746
pH 48 h3)	5.52±0.11	5.70±0.12	0.0004

ADG, average daily gain; LW, live weight; HCW, hot carcass weight.

1) Braford steers (25 months of age) finished with: Suppl, corn silage supplement at 1% of body weight per head/d in addition to *ad libitum* pasture during 120 days; Contr, finished on pasture.

2) Conformation score: JJ = 7, J, U, U2, N, T, A = 1, where higher numbers indicate better conformation, and fat degree (0, 1, 2, 3, and 4), where higher numbers indicate a thicker fat cover, assessed according to the Argentine Classification System (National Meat Department, Resolution J-378/73 of SAGPyA).

3) pH, potential of hydrogen measured 45 min (pH 45 min) and 48 h (pH 48 h) *post mortem* measured at the abattoir in the *longissimus thoracis et lumborum* muscle.

**Table 4:** Meat quality attributes as influenced by finishing treatments measured in thawed longissimus thoracis et lumborum muscle

Variable	Finishing treatment1)		p-value

	Suppl	Contr	
REA (cm^2^)	60.16±7.88	64.54±7.22	0.1233
Marbling score2)	1.77±0.56	1.47±0.55	0.1509
pH	5.55±0.11	5.68±0.15	0.1008
Colour lightness L*	32.82±2.11	31.94±2.71	0.5483
Colour redness a*	16.95±1.14	15.99±1.66	0.2251
Colour yellowness b*	9.38±1.36	8.58±1.87	0.4754
WHC3) (%)	37.75±2.53	31.02±3.52	<0.0001
CL (%)	32.15±2.49	35.51±2.29	0.0006
WBSF (N)	73.58±13.25	62.75±12.77	0.0306
IMF (g/100 g muscle)	3.47±1.24	2.22±1.44	0.0174
Calcium (mM)	0.67±0.16	0.61±0.21	0.5639
Collagen (mg/g)	3.70±0.33	3.66±0.31	0.8861

REA, rib eye area; pH: potential of hydrogen; WHC, water holding capacity; CL, cooking loss; WBSF, Warner-Bratzler shear force; IMF, intramuscular fat content.

1) Braford steers finished with: Suppl, corn silage supplement at 1% of body weight per head/d in addition to *ad libitum* pasture during 120 days; Contr, finished on pasture.

2) Marbling score, (0.0 to 5.0), where 0.0 indicates devoid and 5.0 indicates excess of intramuscular fat (USDA standards).

3) WHC (%) = meat area/total liquid infiltrated area×100.

**Table 5:** Relative levels of calpains and calpastatin mRNA and proteins measured in the *longissimus thoracis et lumborum* muscle

Items	mRNA1)			Protein2)		
	
	Fold change3)	SEM	p-value	Fold change	SEM	p-value
Calpain-1	0.145	0.12	0.0001	0.874	0.06	0.0397
Calpain-2	0.439	0.20	0.0006	0.921	0.10	0.1186
Calpastatin	4.382	0.07	0.0379	2.758	0.12	0.0099
Calpain-1:Calpastatin*	0.031	0.09	0.0100	0.323	0.10	0.0812
Calpain-2:Calpastatin*	0.099	0.15	0.0102	0.328	0.09	0.0742

SEM, standard error of the mean; *GAPDH*, glyceraldehyde-3-phosphate dehydrogenase; *RPLP0*, Ribosomal protein large protein 0.

1) Relative mRNA levels of genes measured by reverse transcription quantitative polymerase chain reaction normalized against *GAPDH* and *RPLP0* reference genes.

2) Protein activity measured by casein zymography (U/g) and normalized against the control group.

3) Fold change was calculated as the relative expression of Suppl group vs Contr group. Fold change greater than 1 denotes increased expression in supplemented group.

* Data values from ratios were transformed using range transformation, this function assigns to the original data the position that each one occupies in the series ordered in ascending order.

**Table 6:** Spearman rank correlation coefficients between calpain:calpastatin ratios at mRNA and protein level in Braford steer *longissimus thoracis et lumborum* muscle

Variable1)	mRNA		Protein
	
	*capn1:cast*	*capn2:cast*	CAPN1:CAST
mRNA			
*capn2:cast*	0.976**	-	-
Protein			
CAPN1:CAST	0.683**	0.664**	-
CAPN2:CAST	0.661**	0.717**	0.717**

1) *capn1:cast*, calpain-1:calpastatin ratio; *capn2*:*cast*, calpain-2:calpastatin ratio at mRNA level; CAPN1:CAST, calpain-1:calpastatin ratio; CAPN2:CAST, calpain-2:calpastatin ratio at protein level.

Data values used for correlation were transformed with range transformation.

** p<0.01 statistical significance.

**Table 7:** Spearman rank correlation coefficients between meat quality characteristics and calpain:calpastatin ratios at mRNA and protein level in Braford steer *longissimus thoracis et lumborum* muscle

Variable	mRNA1)		Protein2)	
	
	*capn1*:*cast*	*capn2*:*cast*	CAPN1:CAST	CAPN2:CAST
WBSF	−0.572**	−0.497**	−0.311	−0.151
CL	0.551**	0.522**	0.573**	0.638**
WHC	−0.715**	−0.717**	−0.707**	−0.650**
HCW	−0.633**	−0.678**	−0.536**	−0.750**
FD	−0.498**	−0.523**	−0.539**	−0.678**
IMF	−0.306	−0.345	−0.565**	−0.608**

WBSF, Warner-Bratzler shear force; CL, cooking loss; WHC, water holding capacity; HCW, hot carcass weight; FD, fat degree; IMF, intramuscular fat content (g/100 g muscle).

1) *capn1*:*cast* and *capn2*:*cast*: calpain (1and 2) to calpastatin ratio at mRNA level.

2) CAPN1:CAST and CAPN2:CAST, calpain (1 and 2) to calpastatin ratio at protein level.

Data values used for correlation were transformed with range transformation.

** p<0.01 statistical significance.
